# The impact of context cues on college students’ purchase behavior for low-carbon products in CBEC

**DOI:** 10.3389/fpsyg.2023.1287235

**Published:** 2023-12-22

**Authors:** Chen Wang, Xiaolong Zhou, Ran Zhang, Yexin Liu

**Affiliations:** ^1^School of Economics and Management, Harbin Institute of Technology (Weihai), Weihai, China; ^2^International Education School, Shandong Polytechnic College, Jining, China; ^3^Department of Public Course, Shandong Polytechnic College, Jining, China

**Keywords:** low-carbon product, context cue, college student, cross-border purchase intention, S-O-R model

## Abstract

**Introduction:**

The purpose of this study was to examine the effects of online shopping context cues (price discount, carbon label, and social commentary) on college students’ trust/perceived risk and cross-border purchase intention (CBPI) for low-carbon products and compare the study between South Korean and Chinese college students. The theoretical framework is established by integrating the S-O-R model and valence framework. The variable of stimuli (S) is the driving force in the purchase behavior that influences the individual organism (O) and its subsequent response (R). Based on this logic, this study draws on the valence framework to hypothesize the effects of online shopping context cues (price discount, carbon label, and social commentary) on college students’ trust/perceived risk and cross-border purchase intention (CBPI) for low-carbon products.

**Methods:**

This study conducted two online experiment-questionnaire studies and one between-subject lab experiment to test the hypotheses.

**Results:**

We found that all three context cues have significant positive effects on trust and negative effects on perceived risk. College students’ trust positively and perceived risk negatively impact college students’ CBPI for low-carbon products. In addition, based on different cultures, languages, and education, this study investigates the moderating effect of college students’ nationality on their significantly different cue processing modes for low-carbon products.

**Discussion:**

The findings provide new insights into better understanding the factors affecting college students’ low-carbon consumption behavior in a CBEC setting and have some practical implications.

## 1 Introduction

Environmental problems caused by CO_2_ emissions to nature are increasing rapidly. To reduce the harm of carbon emissions, various policies are put forward around the world (e.g., South Korean “Green New Deal” and Chinese “30•60 carbon peak and neutrality strategic goals”). Following that, more and more low-carbon products appear in online and offline markets, and global scholars and managers in various fields are paying attention to environmental issues (ElHaffar et al., 2020). Especially, in the field of cross-border e-commerce (CBEC), the emergency of low-carbon products and green consumption represents the inevitable trend (Xue et al., 2022). Thus, improving consumers’ purchase intention for low-carbon products on the CBEC platform can effectively solve global environmental concerns and achieve the dual growth of economic and environmental performance (Wu et al., 2021). At the same time, prior research reveals that college students have emerged as a part of a critical consumer segment globally due to their strong consumption power, their influence in family decision-making, and their leading role in different social groups (van Berlo et al., 2020). Moreover, based on the development of online shopping and the integration of the global economy, the direct or indirect consumption of CBEC by college students is increasing year by year. Therefore, promoting college students’ low-carbon consumption behavior in a CBEC setting would be a significant part in improving the global low-carbon development, which has not received sufficient attention.

Previous CBEC research has not only focused on the positive impact of cross-border transaction volume on the macro-economy but also on the improvement of the shopping experience on CBEC platform microlensing. Recently, with the increasing environmental concerns around the globe, some scholars and managers have tentatively investigated the low-carbon consumption promotion strategy in an online setting (Wu et al., 2021), while the studies on low-carbon consumption on the CBEC platform from a B2C perspective are still in its infancy. Thus, identifying the significant factors on attracting much more online consumers and influencing their low-carbon product purchase intention or behavior finally have become critical for environmental and financial performance purposes for all CBEC platforms (Abdulkarem and Hou, 2021). Based on the Stimulus-Organism-Response (S-O-R) model, previous studies have emphasized that consumers’ internal emotional reactions can be influenced by various external stimuli (e.g., online shopping context cues; Zhu et al., 2020). Owing to the rich and diverse information of the CBEC platform, firms should obtain consumers’ attention to create more business opportunities. It is argued that consumers’ attention always closely correlates with contextual cues. Online shopping context cues are widely used in traditional import/export trade businesses as a representative and effective external stimulus. The effect of online shopping context cues can take place in the attention guidance stage, which subconsciously reinforces consumers’ impressions. Therefore, cross-border online shopping context cues can be used to capture consumers’ attention and thus further affect cross-border purchase intention (CBPI). Considering the high added value and negative image (e.g., high premium, poor quality, and false publicity) of low-carbon products (Wang et al., 2020) and college students’ leading role in different social classes, there is a lack of empirical studies on investigating the antecedents of consumers’ overall perceptions and ultimate purchase intention for low-carbon products in a CBEC setting. To address this research gap, this study attempts to explore the significant effects of online shopping context cues (e.g., price discount, carbon label, and social commentary) on enhancing college students’ overall perception of low-carbon products to achieve a win–win result for all consumers, CBEC platforms, and the environment.

In the same vein, this study also attempts to examine the effect of external stimuli on the ultimate purchase intentions of college students for low-carbon products on the CBEC platform through their overall perception formation. Some previous studies have revealed the explaining role of consumers’ hedonic and functional perceived value (Xiao et al., 2019) and retailer trust (Cui et al., 2019) on the formation mechanism of consumers’ green consumption on the CBEC platform, while according to the valence framework, individuals’ trust and perceived risk are two representative perceptions, which contribute to affect individuals’ final decision-making (Mou et al., 2019a). Moreover, previous studies have not sufficiently explained the influence of online shopping context cues and college students’ trust and perceived risk on their cross-border purchase intention (CBPI) for low-carbon products (Mou et al., 2019b). Therefore, this study attempts to examine how trust and perceived risk build college students’ overall perceptions of college students in relation to cross-border online shopping context cues for the consumption of low-carbon product consumption according to the valence framework.

Furthermore, on account of different habits, languages, education, cultures, lifestyle, and family environment, students constantly focus on different external stimuli of products and online shopping situations (Kim et al., 2017; Li et al., 2017). Hence, college students from different countries (e.g., South Korea vs. China) will elicit different perceptions of online consumption context (Halder et al., 2020). Although both South Korea and China are representative collectivistic countries (Hofstede, 1984), they show different (low-carbon) online shopping behaviors due to their different cultural educations, economy policies, and political environment, which may lead to predictable differences in low-carbon consumption processing in a CBEC setting. In conclusion, this study aims to compare the differential impact of context cues on trust and perceived risk for low-carbon products among South Korean and Chinese college students to unveil their distinct cognitive processes in information processing on CBEC platforms.

Overall, this study makes three main contributions to college students’ low-carbon consumption and CBEC research area. First, we focus on college students’ low-carbon consumption on the CBEC platform and explain the influence mechanism of online shopping context cues on improving college students’ trust and perceived risk for low-carbon products in a CBEC setting. Specifically, this study demonstrates how online shopping context cues for low-carbon product information can be used to reduce individuals’ perceived risk and promote trust so that they can finally transfer to students’ low-carbon purchase intention on the CBEC platform. Second, the valence framework, as a theoretical model, explains the significant effects of trust and perceived risk on CBPI for low-carbon products through CBEC platform. Third, based on different cultures, languages, and education, this study investigates the moderating effect of college students’ nationality on their significant different cue processing modes for low-carbon products in a CBEC setting.

## 2 Theoretical background and hypotheses

### 2.1 S-O-R model in CBEC

Scholars are describing a consistent and clear research frame of the S-O-R model, which has been widely used to examine the significant effects of product properties, situational stimulus, shopping contexts on assessing product/services quality, consumers’ attitude formation, and subsequent purchase decision-making (Zhu et al., 2020). Based on the S-O-R model, physical external stimulus (S) (e.g., music, smell, and even sense of touch) and situational external stimulus (e.g., scenario design, decoration, price, and communication) may trigger individual’s internal reactions such as satisfaction, pleasure, superiority, or even anger (O), which subsequently influence individual’s final intention or behavior for consumption (R) (Zhu et al., 2020).

The S-O-R model has been widely used in analyzing the formation mechanism of individuals’ cognition and perception of (cross-border) online shopping and explaining how the perception subsequently contributes to their final purchase decision-making (Zhu et al., 2020; Liu et al., 2021). For instance, Zhu et al. (2020) developed a conceptual model to explain consumers’ impulse purchase behavior in a CBEC setting based on the S-O-R model. The findings emphasized that an effective product description (including display or content) has a positive influence on consumers’ curiosity, which subsequently stimulates their impulse purchase intention in a CBCE setting. Similarly, several studies have attempted to explore the antecedents of low-carbon product purchase intention in online/offline shopping environments, based on the S-O-R theoretical approaches (Song et al., 2022).

### 2.2 Relation between context cues and individuals’ trust and perceived risk

Previous research has emphasized various antecedents of consumers’ overall perception and purchase intention on low-carbon (green) products (Liu et al., 2016; Abdulkarem and Hou, 2021; Rondoni and Grasso, 2021; Wang et al., 2022). For instance, focusing on the food industry, Rondoni and Grasso (2021) conducted a literature review and revealed that individuals’ demographics and carbon labels’ context type significantly influence consumers’ overall perception and final low-carbon behavior. Abdulkarem and Hou (2021) directly investigated the effects of products’ technological context, organizational context, and environmental context on consumer’s CBEC adoption in China. Wang et al. (2022) provided empirical support with regard to the improvement of SNS users’ trust by enhancing consistency, fit, and impact communication context cues. After sorting out the antecedents in previous research, this study attempts to combine the characteristics of online shopping and low-carbon products and examine the online shopping context cues, namely, price discount, carbon label, and social commentary, that are positioned as representative attributes that influence college students’ trust and perceived for context cues in a CBEC setting in South Korea and China.

1. Price discount refers to a short-term product price reduction for all or specific consumers, which invariably can increase product sales and market share in a short-time effectively (Zhu et al., 2018). Price discount is a basic and useful marketing mix strategy in both online and offline shopping settings on improving individuals’ decision-making process. Giuffrida et al. (2017) revealed that price discounts have a significant effect on consumers’ online purchase decision-making and emphasized this feature by increasing economic stimulus (Tan, 2022). In practice, numerous South Korean and Chinese consumers are sensitive to price fluctuation, which can be verified by *6·18 big sales, the 11·11/12·12 promotion*, and *Christmas/new year big sale* phenomenon.

For low-carbon products, as pleasant surprises, appropriate price reduction can satisfy individuals’ wants for excellent and inexpensive products, reduce the risk perception caused by elevated prices, and then trigger their final green consumption behavior. For cost and technical reasons, low-carbon products always have higher prices (vs. general products). Therefore, if the products are offered by two retailers at different prices in a CBEC setting, which can be predicted to improve individuals’ overall perceived trust and reduce overall perceived risk. Hence, price discount cues are also used to improve consumers’ utilitarian value and reduce their uncertainty perception (Wang et al., 2020).

2. Carbon labels refer to the marker disclose information about carbon emissions and the impact that their production has on the environment, which can help consumers make more sustainable purchase decisions (Rondoni and Grasso, 2021). By choosing the right contents and consumer match, carbon labels can be used for educating potential consumers about the characteristics of firms’ production (Wong et al., 2020). Recently, the implementation of carbon labeling has become a pressing concern for all industries, including CBEC platforms, as they seek to capture consumers’ attention and increase their purchase intention. In such circumstances, some managers and scholars have not only focused on the positive effect of label carbon on the macro-economy but also investigated the improving effects of carbon labels on online/offline shopping environment (Liu et al., 2016). For instance, Grunert et al. (2014) compared the effect on consumers between traditional attributes (e.g., fat content, origin, and price) and food sustainable production attributes (e.g., ecological footprint and health-related aspects). Wong et al. (2020) addressed the carbon footprint of beverage products and investigated consumers’ purchase attitude and behavior toward carbon labels.

The objectivity (e.g., accurate data and authoritative certification) and the independence (e.g., certified by an authority) of carbon labels have made consumers unconsciously persuaded (Grunert et al., 2014). Individuals can receive useful carbon emission or green production information from carbon labels presented online; hence, they can easily understand the green properties of the products on the CBEC platform, which helps to improve the shopping experience and reduce risk perception effectively (Zhao et al., 2018). At the same time, novel, objective, authoritative, and intriguing low-carbon contents can awake individuals’ environmental awareness and enable consumers’ intention on online shopping activities to improve their emotional trust and decrease perceived risk (Lee, 2017).

3. Social commentary is a combination of contents presented by individuals with SNS, which refers to a virtual community where all users can share views, lifestyle, consumption experiences, and ideas with each other (Chen et al., 2021). With the rapid growth of social media, it has attracted more and more college students. At the same time, CBEC platforms and individual retailers have noticed that consumers’ positive commentaries on social media can be a huge boost to product sales and brand improvement; thus, they tend to encourage consumers to actively discuss and share the perception of their consumption experience by writing comments on *Facebook, Instagram, KakaoTalk, Blogs, WeChat, QQ zone BBS*, etc. (Chen et al., 2021).

Previous findings has emphasized that consumers’ social commentary cues influence consumers’ trust effectively in a general online shopping context, that is, the textual commentary is always regarded as a useful evaluation of consumers’ perception of consumption experience, which, moreover, provides a guaranteed set of messages for potential consumers’ low-carbon products consumption processing mode (Wang et al., 2023). Moreover, various social commentaries involve pre-contractual (e.g., carbon emission and product quality description) and post-contractual (e.g., delivery risk, environmental pollution risk, and financial risk) information, which can effectively eliminate individuals’ risk perception in a CBEC online shopping setting (Mou et al., 2019a). Therefore, social commentary cues (e.g., user experience, product rating, and issue discussion for low-carbon products) on SNS should become essential influencing factors on individuals’ overall perception and final purchase behavior. In conclusion, we propose hypotheses as follows:

Hypotheses: Cross-border online shopping context cues (H1a: price discount cues, H2a: carbon label cues, and H3a: social commentary cues) for low-carbon products contribute positively to college students’ trust.

Hypotheses: Cross-border online shopping context cues (H1b: price discount cues, H2b: carbon label cues, and H3b: social commentary cues) for low-carbon products contribute negatively to college students’ perceived risk.

### 2.3 Valence framework and college students’ trust/perceived risk → CBPI relationship

The direct or indirect effects of consumers’ overall perception on their purchase intention/behaviors have been widely emphasized by many studies (Mou et al., 2019b). In some studies, individuals’ overall perception is considered as a multidimensional concept. For instance, Xiao et al. (2019) discuss the significant effect of consumers’ perceived emotional and functional value on online purchase intention. While, based on the valence framework, individuals can format both positive and negative perceptions from a (cross-border) online shopping transactions (Cui et al., 2019), the valence framework explains individual trust and perceived risk as two crucial dimensions in influencing their final decision-making. Specifically, as an estimate of received utility from a product or context cues, individuals’ overall trust always plays an essential role in their purchase decision-making process (Wang et al., 2023). Moreover, individuals’ perceived risk is the negative valence system that detracted from their consumption behavior and is primarily caused by information asymmetry in the online shopping context, especially for low-carbon products on CBEC, which are closely related to misleading communication and high green premium (Mou et al., 2019a).

Previous findings emphasize that individuals tend to increase their net assessment of utility in the general process of consumption (Peter and Tarpey, 1975), that is, in a CBEC setting, college students who received context cues for low-carbon products will compare their benefits in the consumption process with their costs or effort, and the final purchase decision will be made when they get adequate net profit and trust (Ventre and Kolbe, 2020). As a significant factor driving individuals’ online consumption behavior, individuals’ trust is widely investigated that it provides a strong stimulus for their adoption of online/offline shopping (Ventre and Kolbe, 2020), Internet/mobile low-carbon consumption (Liu et al., 2016), and even cross-border shopping consumption process (Har Lee et al., 2011). Thus, we propose the hypothesis as follows:

H4: College students’ trust for context cues contributes positively to their CBPI for low-carbon products in a CBEC setting.

On the contrary, when buying low-carbon products on a CBEC platform, individuals face a lot of information, and their limited knowledge of low-carbon products and relative shopping experience may lead to further misunderstanding of the products or increase uncertainties about retailers’ behaviors (Ding et al., 2018). At the same time, individuals cannot predict the outcome of future transactions on CBEC platforms accurately; thus, it is difficult for college students to assess the profits of CBEC transactions (Mou et al., 2019b). According to the valence theory, individuals tend to reduce disutility in the ordinary shopping process (Peter and Tarpey, 1975); thus, it is difficult for individuals to make consumption decisions in a high risky state. Moreover, some prior studies revealed that individuals’ perceived risk has a negative effect on their purchase intentions and behavior for low-carbon products in e-commerce shopping settings (Chiu et al., 2012; Ventre and Kolbe, 2020). Thus, we propose hypothesis as follows:

H5: College students’ perceived risk for contest cues contributes negatively to their CBPI for low-carbon products in a CBEC setting.

### 2.4 Comparison of south Korean and Chinese college students

Numerous individuals’ characteristics have been investigated in the context of diverse online green shopping process literature (Kim et al., 2017; Jiang et al., 2020). Different individual’s complex information processing for low-carbon product purchasing is always discussed in the sustainable management literature (Halder et al., 2020; Xue et al., 2022). With the proposal of South Korean “*Green New Deal*” and Chinese “*30•60 carbon peak and neutrality strategic goals*,” the environmental awareness of residents in both countries has been considerably improved (Ding et al., 2018), especially college students who received advanced education. Although both South Korea and China are representative collectivistic countries (Hofstede, 1984), they show different (low-carbon) online shopping behaviors due to their different cultural educations, economy policies, and political environment, which may lead to predictable differences in low-carbon consumption processing in a CBEC setting.

Specifically, this study aims to predict this difference from the cultural discrepancy and green value perspectives. First, individuals’ collectivism has been investigated to have a positive effect on their pro-environmental consumption behavior (Lee, 2017; Halder et al., 2020). Although there are some cultural similarities between South Korea and China, which are influenced by Confucianism, South Korean residents show a stronger collectivism in many aspects (Lee, 2017). Therefore, South Korean college students will show extra enthusiasm in low-carbon product information processing. Second, South Korea has adopted the concept of sustainable development as a guiding principle in its environmental policy, law, and education. Compared with China, low-carbon consumption has a better mass base in South Korea (Lee, 2017). Therefore, various information about low-carbon products is more acceptable to the youth people in South Korea and is influenced by them. In conclusion, we suggest that, for South Korean (vs. Chinese) college students’ low-carbon products in a CBEC setting, external cues may lead to stronger stimulating and influential effects. We propose the following hypothesis:

H6: There will be different influence of the context cues on South Korean vs. Chinese college students’ trust and perceived risk in low-carbon product shopping process through CBEC, that is, there will be a stronger influence of the context cues on South Korean (vs. Chinese) college students’ trust and perceived risk in low-carbon product shopping process through CBEC.

**Figure 1:**
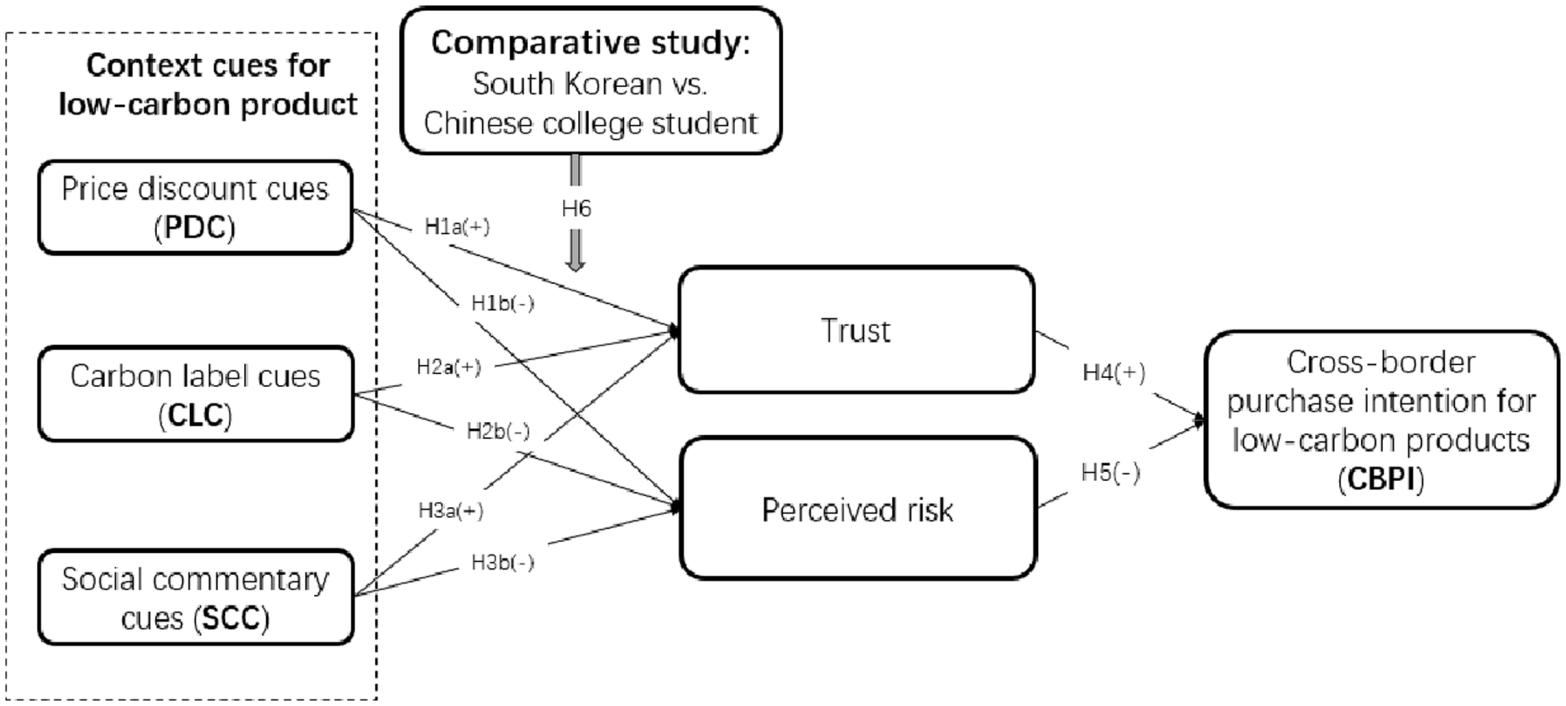
Structural model.

In sum, to examine the hypotheses about “context cues (including price discount, carbon label, and social commentary) → college students’ trust and perceived risk → CBPI for low-carbon products” relationship between South Korea and China, we develop a structural model shown in Figure 1.

## 3 Methods

### 3.1 Measurement development

Based on the hypotheses of the proposed conceptual model, and combining with the object and specific conditions of this research, the measurements used in this study were adapted from previous empirical research (see Appendix). (1) Three context cues: we measured three kinds of context cues by asking students to answer nine questions, three items in each part, adapted from Xiao et al. (2019) and Wong et al. (2020). (2) Students’ trust and perceived risk: this study measured students’ trust with a three-item scale adapted from Ventre and Kolbe (2020) as well as evaluated the perceived risk with a three-item scale adapted from Bianchi and Andrews (2012) and Ventre and Kolbe (2020). (3) Cross-border purchase intention for low-carbon products: based on Chiu et al. (2012), we adapted three items to measure CBPI. Finally, participants’ age, education, gender, and monthly expenditure on online shopping were asked as control variables.

### 3.2 Survey design and sample

The survey instrument was piloted using a number of South Korean and Chinese college students who have experience in cross-border online shopping to assess its appropriateness. In addition, two academics from reputable universities and two online shopping experts examined the questionnaire to assess the face and content validity.

The survey was collated in English and was translated into official South Korean and Chinese languages by two South Korean and two Chinese independent researchers (Hair et al., 2011). After completing the translation, another two professors then back translated the South Korean and Chinese surveys to avoid differences between the two surveys and the English version. This study adopted a between-subject (South Korean college student vs. Chinese college student) design. We collected survey data from South Korean and Chinese college students who have more than one experience in cross-border online shopping (the first question in the questionnaire). To maximize the valid questionnaire collection, both offline and online surveys were distributed. The process had lasted for nearly 7 weeks. All participants were given the survey and asked to recall their recent online shopping experience in a CBEC setting. A total of 817 surveys were distributed, of which a total of 429 were returned (the response rate of 52.6%). After eliminating the questionnaires that did not meet the criteria and were incomplete, a final sample of 352 usable data was used in the final empirical analysis. The degrees mentioned in most samples are junior college (58.2%) followed in second by an undergraduate course, as reported by more than 38.4% of the study sample participants. Detailed descriptive statistics for all students are shown in Table 1.

**Table 1:** Descriptive statistics of all respondent characteristics.

Demographics	Category	Count	Rate (%)
Nationality	South Korea	170	48.3
	China	182	51.7
Gender	Women	172	48.9
	Men	180	51.1
Age (years)	17–18	52	14.8
	19–20	140	39.7
	21–22	148	42.1
	23 or above	12	3.4
Education	Junior college	205	58.2
	Undergraduate	135	38.4
	Graduate course	12	3.4
Monthly expenditure on online shopping ($)	≤ 50	31	8.8
	51–100	94	26.7
	101–150	121	34.4
	151–200	81	23.0
	≥ 200	25	7.1
Total		352	100

## 4 Results

### 4.1 Non-response and common method bias

First, this study divided the independent variables and dependent variables into different parts of the survey to minimize the potential effects of common method variance (Podsakoff et al., 2003). Second, to verify the possible common method bias, based on exploratory factor analysis, we conducted Harman’s single-factor test (Podsakoff et al., 2003). Exploratory factor analysis yielded six factors with eigenvalues above 1.0, which explained 88.8% of the total variance. Thus, there was no serious common method bias in this study. Third, to avoid the non-response bias, we divided all samples randomly into two different groups and tested the differences between their characteristics and demographic data (Armstrong and Overton, 1977). There was no significant difference between the two groups, meaning that non-response bias is not a major issue.

### 4.2 Validity and reliability of measurement model

Using the full sample (*N* = 352) data, we conducted a confirmatory factor analysis with AMOS 18 on six constructs, 18 items. First, to calculate the convergent validity, we linked each item to its corresponding construct and estimated the covariance among all constructs. The fit of model resulted in *χ^2^* = 173.953 (*df* = 120, *p* < 0.05); *RMSEA* = 0.036; *RMR* = 0.042; *GFI* = 0.950; *CFI* = 0.981; and *NFI* = 0.972, the overall fitness of this CFA model was good, and all of the factor loading scores were above 0.60 and *t*-values were higher than 1.96 (see Table 2). Second, six average variance extracted (AVE) were calculated, which are all greater than the 0.60 standard, indicating significant convergent validity. Meanwhile, the correlation between all constructs was all smaller than each construct’s square root of the AVE, revealing the compliance with discriminant validity (Fornell and Larcker, 1981; Hair et al., 2011; see Table 3).

**Table 2:** CFA analysis result (*N* = 352).

Factor	Scale	S. Estimate	*t*	AVE	C.R.
Price discount cues	PDC1	0.949	-	0.896	0.962
	PDC2	0.937	35.793		
	PDC3	0.954	38.478		
Carbon label cues	CLC1	0.925	-	0.842	0.941
	CLC 2	0.907	27.938		
	CLC 3	0.920	28.937		
Social commentary cues	SCC1	0.934	-	0.881	0.951
	SCC2	0.932	32.757		
	SCC3	0.949	34.645		
Trust	Trust1	0.927	-	0.827	0.935
	Trust2	0.894	26.918		
	Trust3	0.907	27.814		
Perceived risk	PR1	0.888	-	0.797	0.921
	PR2	0.923	24.874		
	PR3	0.867	22.509		
Cross-border purchase intention for low-carbon products	CBPI1	0.853	-	0.733	0.892
	CBPI2	0.853	19.029		
	CBPI3	0.863	19.273		

Model fit summary: χ^2^(120) = 173.953; χ^2^/df = 1.450; RMSEA = 0.036; RMR = 0.042; GFI = 0.950; CFI = 0.991; and NFI = 0.972.

**Table 3:** Discriminant analysis result (*N* = 352).

	PDC	CLC	SCC	Trust	Perceived risk	CBPI
PDC	0.946					
CLC	0.294^**^	0.917				
SCC	0.334^**^	0.283^**^	0.938			
Trust	0.546^**^	0.419^**^	0.475^**^	0.909		
Perceived risk	−0.297^**^	−0.365^**^	−0.375^**^	−0.396^***^	0.893	
CBPI	0.259^**^	0.205^**^	0.315^**^	−0.416^**^	0.435^**^	0.856

Diagonal value is the square root of AVE value; table value is correlation coefficient; ^**^p < 0.01; ^***^p < 0.001.

### 4.3 Structural model

**Figure 2:**
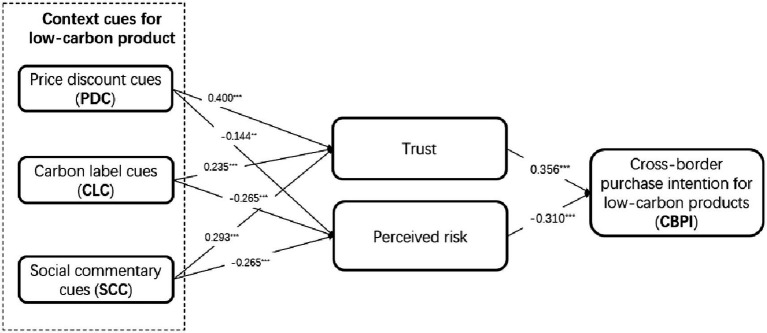
Results of structural analysis (^**^p < 0.05; ^***^p < 0.001).

This study estimated the full sample (*N* = 352) data by the AMOS program to test the structural effect of all six constructs (including three context cues, students’ trust/perceived risk, and CBPI), resulting in an acceptable level of multicollinearity. According to Table 4, model fit showed that *χ^2^* = 182.353 (*df* = 124, *p* < 0.001); *RMSEA* = 0.037; *RMR* = 0.054; *GFI* = 0.948; *CFI* = 0.990; and *NFI* = 0.971, and the overall model fitness was good. Three context cues contributed positively to trust (PDC: *β* = 0.400, *t* = 8.435; CLC: *β* = 0.234; *t* = 5.044; SCC: *β* = 0.293, *t* = 6.224), and H1a–H3a were all supported. Furthermore, three context cues contributed negatively to perceived risk (PDC: −0.144; *t* = −2.612; CLC: *β* = −0.265, *t* = −4.797; SCC: *β* = −0.265; *t* = −4.763), and the resulting H1b–H3b were all supported. As speculated, H4 and H5 were also supported in that trust contributed positively and perceived risk contributed negatively to CBPI for low-carbon products (Trust: *β* = 0.356; *t* = 6.412; Perceived risk: *β* = −0.310; *t* = −5.539; see Table 4; Figure 2). In addition, we tested all control variables and found no significant influence.

**Table 4:** Structural analysis results (*N* = 352).

Pathway		S. Estimate	S. E.	*t*	Results
H1a	PDC → Trust	0.400	0.038	8.435^***^	Accepted
H2a	CLC → Trust	0.235	0.042	5.044^***^	Accepted
H3a	SCC → Trust	0.293	0.038	6.224^***^	Accepted
H1b	PDC → Perceived risk	−0.144	0.038	−2.612^**^	Accepted
H2b	CLC → Perceived risk	−0.265	0.044	−4.797^***^	Accepted
H3b	SCC → Perceived risk	−0.265	0.038	−4.765^***^	Accepted
H4	Trust → CBPI	0.356	0.053	6.412^***^	Accepted
H5	Perceived risk → CBPI	−0.310	0.062	−5.539^***^	Accepted

Model fit summary: χ^2^(124) = 182.353; χ^2^/df = 1.471; GFI = 0.948; CFI = 0.990; NFI = 0.971; RMSEA = 0.037; RMR = 0.054; ^**^p < 0.01.^***^p < 0.001.

### 4.4 Moderating effect of nationality

Following the structural analysis, H6 explained the different effects of context cues → trust and context cues → perceived risk relationships of South Korean and Chinese college students’ low-carbon consumption process on CBEC, and multi-group comparison analysis by AMOS was performed. Theoretically, there are different rules for a minimum sample size for SEM, for example, a minimum sample size of 100 or 200, 5 or 10 observations per estimated parameter, and 10 cases per variable (Wolf et al., 2013). The larger sample size can obtain better estimates of parameters and chi-squared probabilities. However, previous research indicates that the marginal sample sizes can also be associated with a satisfactory fit, low Type-I error rates, and stable model parameters (Sideridis et al., 2014). Although the sample size for this study is not idealistic, it meets the rules of the minimum sample size for SEM. Therefore, the structural analysis for two separate groups is feasible (Li and Jacobucci, 2022; Alrawad et al., 2023).

**Figure 3:**
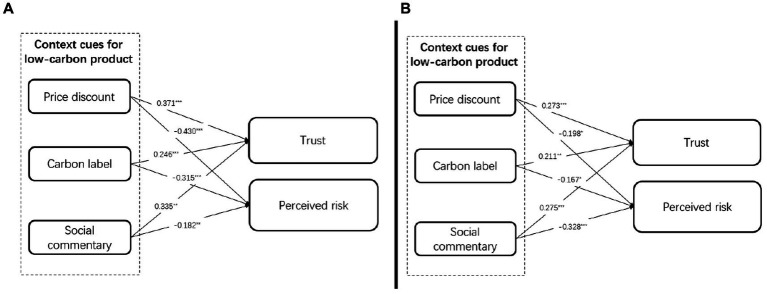
Results of structural analysis for different student groups. **(A)** South Korean college student group. **(B)** Chinese college student group.

First, we divided two separate groups: South Korean college student group vs. Chinese college student group. The results in Table 5 show that, in the South Korean student group, all three context cues contributed positively to trust (PDC: *β* = 0.371, *t* = 5.496; CLC: *β* = 0.246, *t* = 3.694; SCC: *β* = 0.335, *t* = 4.898). Moreover, all three context cues contributed negatively to perceived risk (PDC: *β* = −0.430, *t* = −6.183; CLC: *β* = −0.315, *t* = −4.608; SCC: *β* = −0.182, *t* = −2.651). Similarly, in the Chinese consumer group, all three context cues contributed positively to trust (PDC: *β* = 0.273, *t* = 3.621; CLC: *β* = 0.211, *t* = 2.867; SCC: *β* = 0.275, *t* = 3.760), and all three context cues contributed negatively to perceived risk (PDC: *β* = −0.198, *t* = −2.546; CLC: *β* = −0.167, *t* = −2.189; SCC: *β* = −0.328, *t* = −4.223). In conclusion, except “SCC → Perceived risk” path (which is contrary to prediction), the *β* value of each path in the South Korean student group was all greater than those in the Chinese student group (see Figure 3).

**Table 5:** Structural analysis for two separate groups.

Pathway	South Korean college student (Group A, *n*1 = 170)		Chinese college student (Group B, *n*2 = 182)	
	St. Path coefficient	*t*	St. Path coefficient	*t*
PDC → Trust	0.371	5.496^***^	0.273	3.621^***^
CLC → Trust	0.246	3.694^***^	0.211	2.867^**^
SCC → Trust	0.335	4.898^**^	0.275	3.760^***^
PDC → Perceived risk	−0.430	−6.183^***^	−0.198	−2.546^*^
CLC → Perceived risk	−0.315	−4.608^***^	−0.167	−2.189^*^
SCC → Perceived risk	−0.182	−2.651^**^	−0.328	−4.223^***^

^*^p < 0.05; ^**^p < 0.01; ^***^p < 0.001.

Finally, to test the moderating effect of college students’ nationalities on context cues → trust/perceived risk relationship, △*χ^2^* (*df* = 1) value between the constraint model and the free model in each path is shown. The results revealed that three differences were significant at *a* = 0.05 level (PDC → Trust: △*χ^2^* = 4.3 > 3.84; CLC → Trust: △*χ^2^* = 6.2 > 3.84; SCC → Perceived risk: △*χ^2^* = 4.6 > 3.84), while no significant differences were found in “SCC → Trust: △*χ^2^* = 0.1 < 3.84; PDC → Perceived risk: △*χ^2^* = 1.1 < 3.84; CLC → Perceived risk: △*χ^2^* = 2.3 < 3.84 paths. Thus, H6 was partially supported (see Table 6).

**Table 6:** Moderating role of college students’ nationality.

Pathway		*χ^2^*		△*χ^2^* (*df* = 1)	*p* value	Results
		Free model	Constraint model			
H6a	PDC → Trust	330.3 (*df* = 248, *p* < 0.001)	334.6	4.3	<0.05	Accepted
H6b	CLC → Trust		336.5	6.2	<0.05	Accepted
H6c	SCC → Trust		330.4	0.1	n.s.	Rejected
H6d	PDC → Perceived risk		331.4	1.1	n.s.	Rejected
H6e	CLC → Perceived risk		332.6	2.3	n.s.	Rejected
H6f	SCC → Perceived risk		334.9	4.6	<0.05	Rejected

Standard △*χ*^2^_(df = 1)_ = 3.84.

## 5 Conclusion and discussion

This study examines the “three context cues for low-carbon products → college students’ overall perception → CBPI for low-carbon products” relationship, as we as the moderating effect of college students’ nationality on the relationship between three types of contextual cues and trust/perceived risk. First, the findings revealed that all three context cues (price discount, carbon label, and social commentary) have significant positive effects on trust and negative effects on perceived risk. Moreover, trust positively and perceived risk negatively impact college students’ CBPI for low-carbon products, supporting H1–H5. These results are relevant, as a basic and useful marketing mix strategy, and the significant effects of price discount/information disclose (carbon label)/SNS communication (social commentary) on improving individuals’ decision-making process are widely investigated (Giuffrida et al., 2017; Wang et al., 2020; Chen et al., 2021; Tan, 2022). Specifically, some previous studies suggest that the price of a product will lead to consumers’ doubts about the quality of the product (Keller et al., 2022; Tan, 2022). However, in this study, consumers show a positive attitude on price discount, that is, the consumers believe that lower prices represent stronger firm competence, greater production scale, and higher technology in a CBEC online shopping setting. In the same vein, appropriate carbon labels can be also used for educating potential consumers about the characteristics of firms’ production (Wong et al., 2020). Compared to the traditional product consumption environment (Grunert et al., 2014; Wong et al., 2020), online low-carbon consumption contains more uncertain factors (Zhao et al., 2018). Therefore, according to our findings, the objectivity and independence of carbon labels can help consumers improve the shopping experience and reduce risk perception effectively. At the same time, social commentaries including pre-contractual and post-contractual information can effectively eliminate individuals’ risk perception in a CBEC online shopping setting (Mou et al., 2019a).

Furthermore, as our experimental findings reveal that, in the South Korean (vs. Chinese) college student group, all three context cues for low-carbon products have a significant stronger impact on trust; moreover, price discount and carbon label cues have stronger effects on perceived risk as assumed. Thus, H6 is partially supported. Some previous studies have investigated various psychological and behavioral differences between South Korea and China in general online/offline consumption process (Chu et al., 2019; Fan et al., 2022; Wang et al., 2022). Nationality and cultural differences are rarely addressed in investigating consumers’ low-carbon consumption behavior (Wang et al., 2023). While, as we investigate in this study, due to different cultural educations, economy policies, and political environment, South Korean and Chinese college students have shown predictable differences in low-carbon consumption processing in a CBEC setting; therefore, it is particularly important to study the differences in low-carbon consumption characteristics through the lens of regional and cultural differences (Shavitt and Barnes, 2020; Fan et al., 2022).

## 6 Implications and limitations

### 6.1 Theoretical contributions

First of all, this study augments advances in the S-O-R model with insights from low-carbon consumption and CBEC literature to theorize how college students process contextual cues for low-carbon products in a CBEC setting. In terms of the positive impact of online shopping context cues on trust by price discount cues rank first, that by social commentary cues second, and that by carbon label cues rank last. Moreover, antecedents’ negative impact on perceived risk is listed as carbon label, social commentary, and price discount cues in order.

Second, the results reveal that trust positively and perceived risk negatively impact college students’ CBPI for low-carbon consumption in a CBEC setting, which is not sufficiently valued by existing CBEC or green consumption literature. This study contributes to existing studies by extending the valence framework and examining the significant effect of positive (trust) and negative (perceived risk) perception on college students’ CBPI for the increasing low-carbon products. This study also responds to the calls for more research on reducing consumers’ attitude–behavior gap during low-carbon consumption in an online shopping context (ElHaffar et al., 2020).

Third, few studies have focused on potential differences in low-carbon consumption behavior between South Korean and Chinese college students (Lee, 2017). The findings reveal that the nationality of college students partially moderates the “context cues → trust” and “context cues → perceived risk” relationships. As a branch of green consumption research, the main contribution of this study is that it extends cultural discrepancy and green value perspectives with a better prediction power for comparing the differences between South Korean and Chinese college students’ low-carbon behavior in a CBEC setting. In addition, contrast to our hypothesis, the influence of social commentary cues on perceived risk is greater in Chinese (vs. South Korean) student groups. A possible explanation for the results contrary to H6 may be that, with the development of digital technology and lifestyle changes due to COVID-19, Chinese college students pay more attention to online life and online social interaction. Therefore, Chinese (vs. South Korean) college students are more likely to be influenced by social commentary cues, which leads to positive perception and final purchase behavior.

### 6.2 Managerial implications

This study extends the study of low-carbon consumption in a CBEC setting by investigating three representative context cues as a driver of college students’ CBPI for low-carbon products associating with trust and perceived risk. The findings provide several constructive managerial implications for CBEC platforms and our society. Furthermore, this study also attempts to discuss the effective measurements and managerial implications to guide college students’ low-carbon product consumption behavior from the cross-border consumption behavior perception and to arouse people’s awareness of low-carbon development around the globe. Growing cross-board trade volume, increasingly stringent CBEC environment, and ever-increasing environmental aware consumers have brought CBEC firms’ attention to external context cues and environmental issues (Wu et al., 2021). To enhance college students’ overall perception of the information and quality of various low-carbon products, as well as to strengthen their online low-carbon consumption patterns, CBEC platforms and individual sellers should develop a sophisticated marketing mix that aligns with the effective online shopping environment.

Specifically, according to the analysis results of “context cues → college students’ trust and perceived risk → CBPI for low-carbon products” relationship, CBEC platforms and individual sellers have a chance to use the appropriate information with regard to price discount, carbon label, and social commentary cues, which can enhance trust and reduce perceived risk, when promoting low-carbon products in a CBEC setting for college students. First, analysis results reveal that college students pay main attention to products’ economic cost, and price discount plays the most crucial role in their online low-carbon consumption behavior. Thus, for low-carbon products that meet the standards, the platform giving appropriate subsidies (e.g., discounts, point redemption, and group building) preference should be very feasible. Second, social commentary, as an effective measure for increasing college students’ trust and reducing perceived risk in online shopping, should be encouraged by CBEC platforms. With the rapid development of various kinds of social media (e.g., W*eChat*, *KakaoTalk*, *and TikTok*), CBEC platforms should invite college students to actively discuss and share the perception and evaluation of their low-carbon consumption experience, which can be provided as a set of useful messages for potential consumers’ low-carbon information processing. Third, carbon label is one of the most widely used communication methods in low-carbon product online shopping context cues (Rondoni and Grasso, 2021), whereas, due to rampant contents (e.g., greenwashing and false propaganda), carbon label cues show relatively weak impact on college students’ low-carbon consumption (Wong et al., 2020). Thus, in the era of digital marketing, the innovation of carbon label strategy is also an essential issue that every CBEC platform must face.

In addition, the comparative study on South Korean and Chinese college students’ analysis results indicates that CBEC managers in China require distinct marketing approaches from those of South Korea. As a result, communication strategy for Chinese young people requires additional collective approaches. For instance, highlighting students’ low-carbon dealing with their social members should be effective. Moreover, for South Korean college students, it requires more utilitarian approaches. For instance, real and effective price discount coupons and low-carbon communication content will be useful and effective. Simultaneously, it necessitates collaborative efforts from CBEC platforms, governments, and society to standardize online trading practices, ensure information authenticity, enhance the low-carbon education of young individuals, and foster enthusiasm for low-carbon consumption among all residents.

### 6.3 Limitations

This research also has limitations that provide advice for the future study. First, although this study used valid and reliable measurements adapted from prior studies, and advice from 45 pre-tested participants, in terms of a large population base, several important independent variables need to be explored and larger-scale exploratory research needs to be conducted in future. Second, various factors such as incentive programs might also have an impact on CBPI. Further studies are necessary to control other factors on the overall perception of college students to explore the impact of online shopping context cues on CBPI. Third, in this study, the questionnaires were mainly randomly distributed in Shandong Province (the third largest economic region in China) and Gyeonggi Province (the first largest economic region in South Korea). In view of the significant differences in economic, educational, and population levels among the regions in China and South Korea, moreover to generalize the results, participants from more provinces should be recruited.

## Data availability statement

The datasets presented in this article are not readily available because it is stated to the respondents that the data will only be used for academic research and will not be made publicly available. Requests to access the datasets should be directed to victorwang1921@163.com.

## Ethics statement

Ethical review and approval was not required for the study on human participants in accordance with the local legislation and institutional requirements. Written informed consent from the patients/participants or patients/participants legal guardian/next of kin was not required to participate in this study in accordance with the national legislation and the institutional requirements.

## Author contributions

CW: Writing – original draft. XZ: Writing – original draft. RZ: Writing – original draft. YL: Writing – review & editing.

## Appendix

**Table:**

Scale items in survey (seven-point Likert)				
Variable		Source	Items	Cronbach’s α
Content cues	Price discount cues	Nagadeepa et al. (2015); Xiao et al. (2019)	(**PDC1**) There are many forms of price discount for low-carbon products on CBEC platforms. (**PDC2**) The price discount of low-carbon products on CBEC platforms is strong. (**PDC3**) Imported low-carbon products’ price discount on a cross-border e-commerce platform is frequent.	0.963
	Carbon label cues	Wong et al. (2020); Xiao et al. (2019)	(**CLC1**) There are many forms of carbon label for low-carbon products on CBEC platforms. (**CLC2**) The carbon label can help me to select the right low-carbon products. (**CLC3**) Products’ carbon label contents on CBEC platform are useful to me.	0.941
	Social commentary cues	Animesh et al. (2011); Zhang et al. (2014)	(**SCC1**) I get a good impression of other residents in the virtual world. (**SCC2**) I develop good social relationships with other community members. (**SCC3**) I am willing to share my own shopping experiences with my friends.	0.956
Trust		Ventre and Kolbe (2020)	(**Trust1**) I feel safe in purchasing low-carbon products on the CBEC platform that protects my privacy. (**Trust2**) I am confident in buying low-carbon products from secured CBEC platform. (**Trust3**) Overall, shopping on the CBEC platform delivers me good value and trust.	0.934
Perceived risk		Bianchi and Andrews (2012); Ventre and Kolbe (2020)	(**PR1**) I feel that purchasing low-carbon products from the CBEC platform involves a high degree of risk. (**PR2**) I feel the risk associated with purchasing low-carbon products from CBEC platform is high. (**PR3**) I am exposed to many transaction risks if I purchase low-carbon products online from the CBEC platform.	0.921
Cross-border purchase intention for low-carbon products		Chiu et al. (2012)	(**CBPI1**) I will consider purchasing imported low-carbon products on the CBEC platform. (**CBPI2**) I am willing to buy imported low-carbon products on the CBEC platform. (**CBPI3**) I will buy imported low-carbon products through the CBEC platform within 6 months.	0.892
